# Low-cost system for analysis pedestrian flow from an aerial view using Near-Infrared, Microwave, and Temperature sensors

**DOI:** 10.1016/j.ohx.2023.e00403

**Published:** 2023-02-14

**Authors:** M. Mejia-Herrera, J.S. Botero-Valencia, D. Betancur-Vásquez, E.A. Moncada-Acevedo

**Affiliations:** a Grupo de Investigación en Tecnologías Emergentes Sostenibles e Inteligentes (GITESI), Institución Universitaria de Envigado, Medellín, Colombia; b Grupo de Sistemas de Control y Robótica (GSCR), Instituto Tecnológico Metropolitano, Medellín, Colombia

**Keywords:** Pedestrian flow, Low-cost, 3D printing, Sensor fusion, Near-Infrared, Microwave, Temperature sensors

## Abstract

The use of IoT systems that support the construction of smart cities is a global trend that directly affects the quality of life of citizens. for vehicular and pedestrian traffic, the detection of living beings and especially of humans, is a way of quantifying different variables pertinent to the improvement of roads, traffic flows, frequency of visits, among others. the implementation of low-cost systems that do not involve high-processing systems makes the solutions more scalable at a global level. The data acquired by this type of device offers advantages to the different entities in statistics and public consultations, thus contributing to their growth. In this article, an assistance system for the task of pedestrian flow detection is designed and constructed. It integrates strategically located arrays of sensors to detect the direction and general location, which include microwave sensors to detect motion, and infrared presence sensors. The results demonstrate that the system manages to establish the direction of flow of the individual and laterally of the displacement and differentiation between humans and objects for assistance to other systems of counting or analysis of pedestrian flow.

## Graphical abstract

**Figure:**
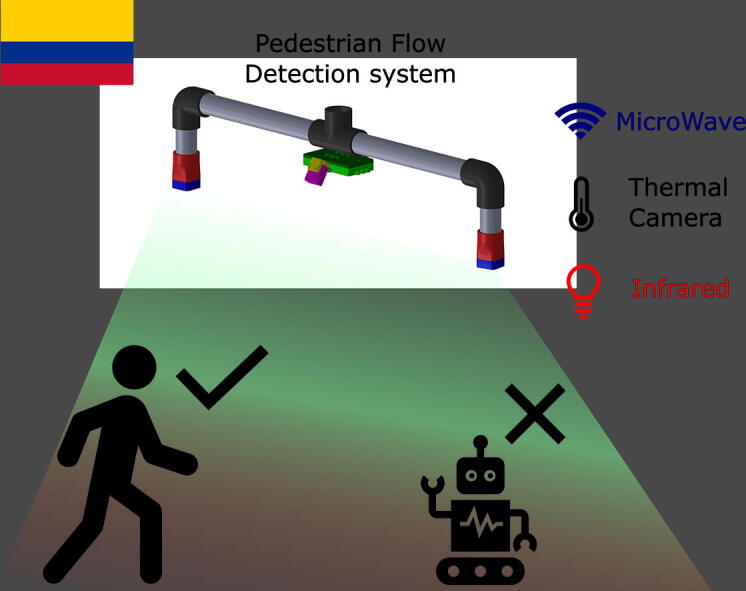

## .

**Specifications table**

**Table:** 

**Hardware name**	*Low-cost system for analysis pedestrian flow from an aerial view using Near-Infrared, Microwave, and Temperature sensors*
**Subject area**	*The designed system allows assistance for pedestrian flow detection without invading personal privacy, this will entitle its use in related research and its application in buildings or smart cities.* • *Engineering and material science* • *Educational tools and open source alternatives to existing infrastructure*

**Hardware type**	*Through the fusion of the information acquired using different sensors, the system can discrssssssiminate the presence of a human being and transmit the data to Wireless Network for remote monitoring or analysis.* • *Field measurements and sensors* • *Internet Of Things)*

**Closest commercial**	*PTZ camera with Human being AI detection.*

**Open source license**	*Creative Commons Attribution-ShareAlike license*

**Cost of hardware**	$*151.01 USD***(Bill of materials).**

**Source file repository**	https:/doi.org/10.17605/OSF.IO/5ND9Y

## Hardware in context

Due to the rapid increase of global warming and its associated problems, some worldwide strategies such as the SDGs (Sustainable Development Goals) have been conceived to promote the development of mechanisms or technology that aims to preserve biodiversity and improve human life quality [1]. In the implementation of this type of people counting technology, it can be classified into two segments as device-based [2], [3], [4] and device-free approaches, which depends on the use of devices carried by the pedestrian, or if they are implemented in the infrastructure [5]. As an example of this, it can be found commercial and academic proposals that serve to monitor and obtain data from a zone [6], One of them is pedestrian flow monitoring systems. These kind of technologies facilitate the management of resources and reduce human and economic losses caused by work and traffic accidents [7]. At the same time, it is a key tool for the analysis of the behavior of a city, market analysis, as well as crowd monitoring for disaster prevention [8]. The determination in the use of Device-Free sensors, allows the independence of the system in the collection of data for the identification of the passer-by. The need to establish and depend on equipping the pedestrian with a device segments the type of public to be accounted for [9], [10].

The detection and counting of pedestrians becomes necessary in the automation of traffic systems in the different streets. Being able to discriminate between humans, animals and other objects that emit radiation, therefore and depending on, the application could prevent the passage of vehicles in pedestrian crossings on the street in smart cities [11], [12]. A valid segmentation when crossing a dense amount of information could discriminate if is many people crossing or there is only one person in the detection range [13]. In this work, a pedestrian counting database is taken using sensory devices of thermal camera technology, infrared and microwave presence sensors. The data was taken in regions of common pedestrian passage and then validated with respect to a high resolution digital camera.

## Hardware description

This project aims to design an integrated system for assisting counting people through thermal, PIR (Presence Infrared), and microwave sensors by the acquisition of meaningful information during the pedestrian monitoring process. The system integrates embedded devices that allow the publication of data to the cloud with additional information such as global positioning. And with the possibility of adding other sensors such as climate or lighting to relate pedestrian flow with environmental variables or specific situations by using I^2^C communication. Unlike some monitoring systems that use RGB (Red, Green, and Blue) or depth [14], [15], [16], [17], [18] cameras. In some countries, the use of recognition of people through artificial vision by digital cameras is a complicated issue due to the processing of personal data, to the point of being illegal. The European Commission has banned the use of artificial intelligence to recognize people in public places [19]. The implementation of sensory systems for pedestrian counting offers advantages in Smart cities to know traffic trends in strategic places. Counting pedestrians without abolishing people’s privacy offers a viable alternative for interested entities [20]s. The proposed system assists such a task without violating people’s privacy since it is impossible to store biometric parameters.

As seen in articles such as [17], [21], [22], one of the approaches to avoiding capturing facial information of an individual and protecting its safety is to use depth cameras from an aerial perspective. However, these approaches may present flaws in the presence of objects similar to individuals, or those that present distributions similar to the Mexican hat wavelet, which can confuse a system and make it consider them as human beings. The proposed design serves as a support for these systems by reducing the mismatch caused by objects, robots, or machinery. Distinguishing when it comes to an object, a robotic system, or a human being. In the configuration present in this article, The system captures the data from an aerial perspective. However, its construction and design allow it to be installed in different planes (X, Y, Z) or vary the relative position of the sensors to adjust to various studies or situations.

• Low cost System
• CAD (computer aided design) and 3D manufacturing which facilitates its replication and modification
• Bluetooth, Wi-fi and Lora communication, which enables it for IoT (Internet of Things) environments
• Distinguish between humans and machinery, for collaborative robotics environments
• I^2^C connectivity to add more sensors or modify settings

The algorithm of the system was codified on arduino IDE using C code for ESP-32 board, and works as follows

First, the algorithm develops a system initialization setup, which specifies the variables and connection pins of each hardware component involved in the system. And start Serial, Bluetooth, and I2C communications for data transmission on and off the system.

Second, (1) a count variable that starts at zero and increases at each iteration is created to help filter the activation frequency at the earlier explained movement() function. Then, the system reads MW and PIR digital pins and applies a delay of 50 ms for the correct sensor readings.

Third, the variables tprev1 and tprev2 store the previously obtained information of “Thermal1” and Thermal2 respectively, or zero by default. While variables Thermal1 and Thermal2 store the readings of the average temperature acquired by each thermal camera, in this way with the present and previous state, the temperature detection activates within the movement function by comparing their difference with a threshold of 0.5 °C. This value is used since the temperature is a variable of slow change in natural conditions and modifications of 0.5 °C degrees are abrupt for such frequent readings thus, they are considered sources of heat external to the environment.

**Fig. 1:**
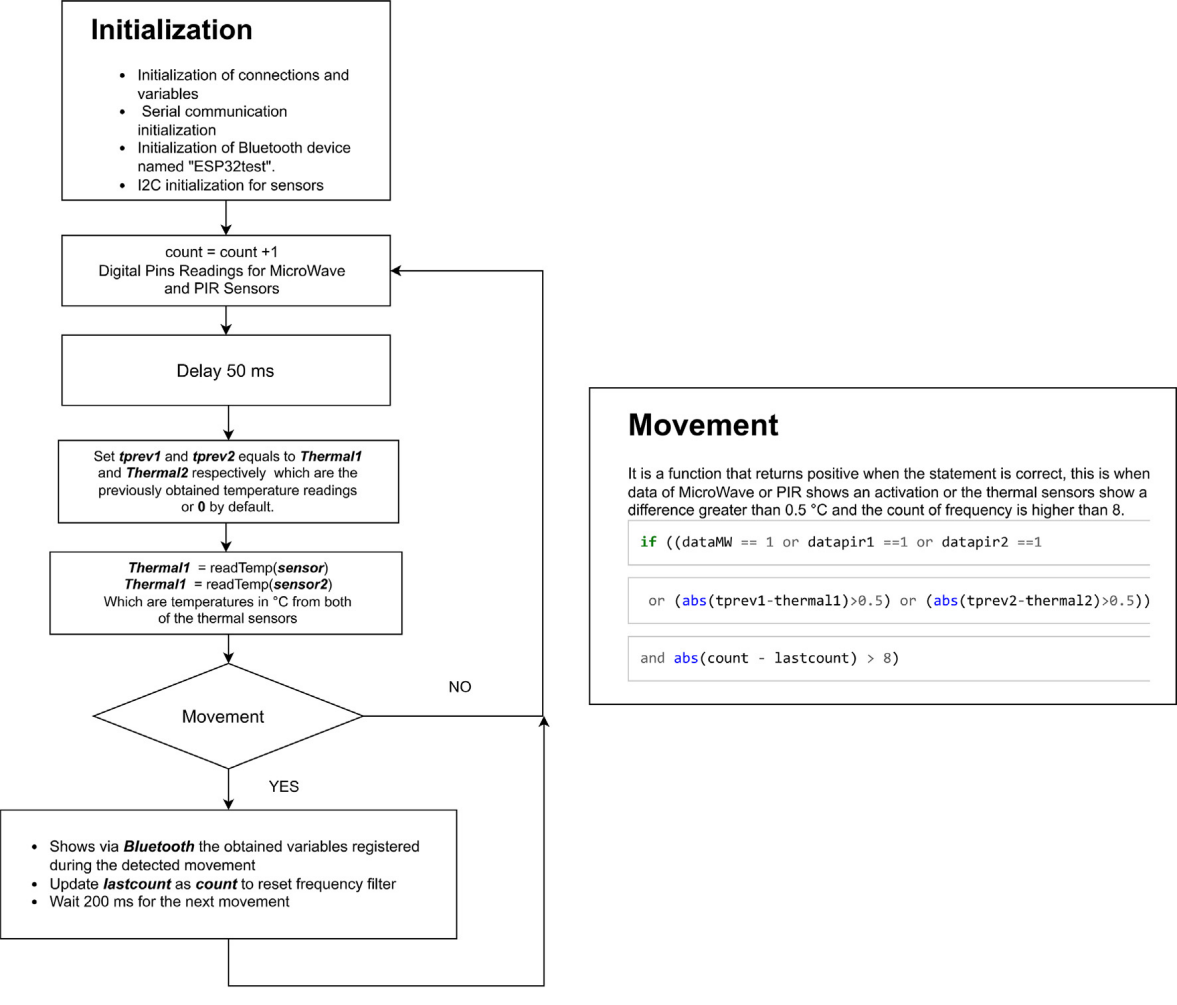
Acquisition code Flowcharts.

Finally, after updating tprev1 and tprev2, the system reads the new Thermal1 and Thermal2 values and calls the movement() function for pedestrian detection based on the frequency filter activations, the activations of each sensor, and the thermal differences. If no movement has been detected, the count variable increments, and the cycle of readings and verification of the movement function repeats. Otherwise, the lastcount value is updated to restart the motion frequency filter and wait 200 ms to repeat the process from (1), as shown in the Fig. 1.

## Design files summary

**Table:** 

**Design filename**	**File type**	**Open source license**	**Location of the file**
MicroWaveFront	STL	CC-BY-4.0	https://osf.io/9tkbz
MicroWaveBack	STL	CC-BY-4.0	https://osf.io/am7wc
PIRFront	STL	CC-BY-4.0	https://osf.io/hxkna
PIRBack	STL	CC-BY-4.0	https://osf.io/csyxk
ThermalFront	STL	CC-BY-4.0	https://osf.io/usz6x
ThermalBack	STL	CC-BY-4.0	https://osf.io/3ysnm
MicroWaveFront	STEP	CC-BY-4.0	https://osf.io/me4wu
MicroWaveBack	STEP	CC-BY-4.0	https://osf.io/eykqr
PIRFront	STEP	CC-BY-4.0	https://osf.io/wbke5
PIRBack	STEP	CC-BY-4.0	https://osf.io/z58ju
ThermalFront	STEP	CC-BY-4.0	https://osf.io/ygjc9
ThermalBack	STEP	CC-BY-4.0	https://osf.io/tdr2p

## Bill of materials summary

This section presents in detail the quantities, prices and references of the devices and other hardware involved in the system construction process.

**Table:** 

Designator	Component	Amount	Cost per unit USD	Total cost USD	Source of materials	Material type	
DFR0478	ESP32 Fire-Beetle	1	$6.90 USD	$6.90 USD	shorturl.at/klnr4	-Composite	
SEN0192	Microwave Sensor V2.0	1	$8.90 USD	$8.90 USD	shorturl.at/gqs37	-Composite	
SEN0171	Digital PIR sensor	2	$4.90 USD	$9.8 USD	shorturl.at/kMQ49	-Composite	
AMG8833	IR Thermal Camera	2	$44.95 USD	$89.9 USD	shorturl.at/aopwC	-Composite	
3/4” PVC Pipe	PVC Pipe	4ft	$5.98 USD	$11.96 USD	shorturl.at/apU05	-Structural	
3/4” PVC Tee	PVC Tee	1	$0.96 USD	$0.96 USD	shorturl.at/cEFNT	-Structural	
3/4” PVC Elbow	PVC Elbow	2	$1.29 USD	$2.6 USD	shorturl.at/mQX08	-Structural	
M3 hardware	M3 Assortment Kit	1	$15.99 USD	$15.99 USD	shorturl.at/hqZ25	-Structural	
M2 Screws	M2 *50 mm screws	8	$0.15 USD	$1.2 USD	shorturl.at/glOT6	-Structural	
M2 nuts	M2 nuts	8	$0.35 USD	$2.8 USD	shorturl.at/suvxz	-Structural	

Total				$151.01 USD			

## Build instructions

The system, as previously mentioned consists of Two 8-pixel thermal cameras, two infrared presence sensors distributed linearly in the main direction of the pedestrian flow, a microwave sensor, and a Fire-beetle ESP32 micro-controller. The following are the steps in an orderly manner for the construction of the described system, and using the numbers of the pieces shown in Fig. 4:

1. Insert the microwave sensor into the front housing of the microwave sensor (1), considering its orientation and position. As a guide, you can use the rounded and squared perforations that indicate the location of the two LEDs of the microwave sensor, and the sensitivity adjustment potentiometer.
2. Fix the back of the microwave sensor housing (2) inserting an 2 cm M3 screw into each of the four perforations found in the corners of the case. Note that the back has the necessary shape to embed the respective M3 nuts.
3. Take the PIRFront piece (4), and introduce the PIR sensor with the Fresnel lens (white hemispherical part) into the perforation of the housing presented in the 2. Note: there are two possible sensor orientations. However, only one allows the connection cable insertion through the PIRBack part (3). Please consider this fact when assembly.

**Fig. 2:**
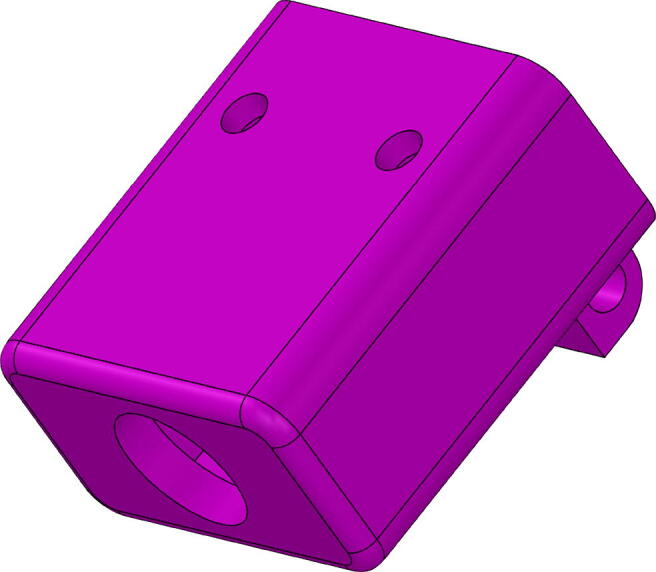
PIRFront piece (4), frontal part of the PIR sensor case.
4. Proceed to insert the 2.5 cm M3 screws to fix the assembly. Keep in mind that the cover has the space to embed the nuts. It is recommended to take special care in embedding the nuts since failures in the printing stage can result in the breakage of the piece during its insertion and fastening. Repeat this procedure for both sensors.
5. The MicroWave sensor (1) case has four groups of extrusions, distributed on the center of each side. Insert in these the PIR assembly (3), using the extrusions to create a hinge with both parts as shown in the 3, by introducing a screw M3 as the axis of the hinge and securing it with its respective nut. This process must be repeated for both sensors. And the Fresnel lens must be pointed in the same direction as the front of the microwave sensor (the face that has the LEDs).
6. For the chassis of the system, a 3/4 inch PVC pipe with its respective accessories was used. For the thermal camera assembly, the AMG8833 is inserted into the ThermalFront (10) and fixed with the ThermalBack (5) using 4 cm M2 screws and their respective nuts. It is advisable to make the connections of this sensor before putting the screws to facilitate the construction process, in addition to using a cable of at least one meter in length.
7. The thermal camera wiring must be inserted through the pipes (6,7,9), and fix the piping using PVC glue. The actual settings and camera orientation is aligned with the rest of the system to have an orthogonal view of the floor. The 10 cm (6) tubes are inserted into the back of the thermal camera housing (5), followed by the PVC elbow (7) and the tube subsequently from the 30 cm tube (9), these pipes will serve as a support, and as a conduit for carrying the cameras wiring. Finally, join the assembly of the thermal cameras described in the previous step using a T of PVC see 4, the free hole serves to couple with another anchoring system, taking into account the wiring.
8. Finally, the wiring of the system should be carried out following the schematic of the Fig. 5.

**Fig. 5:**
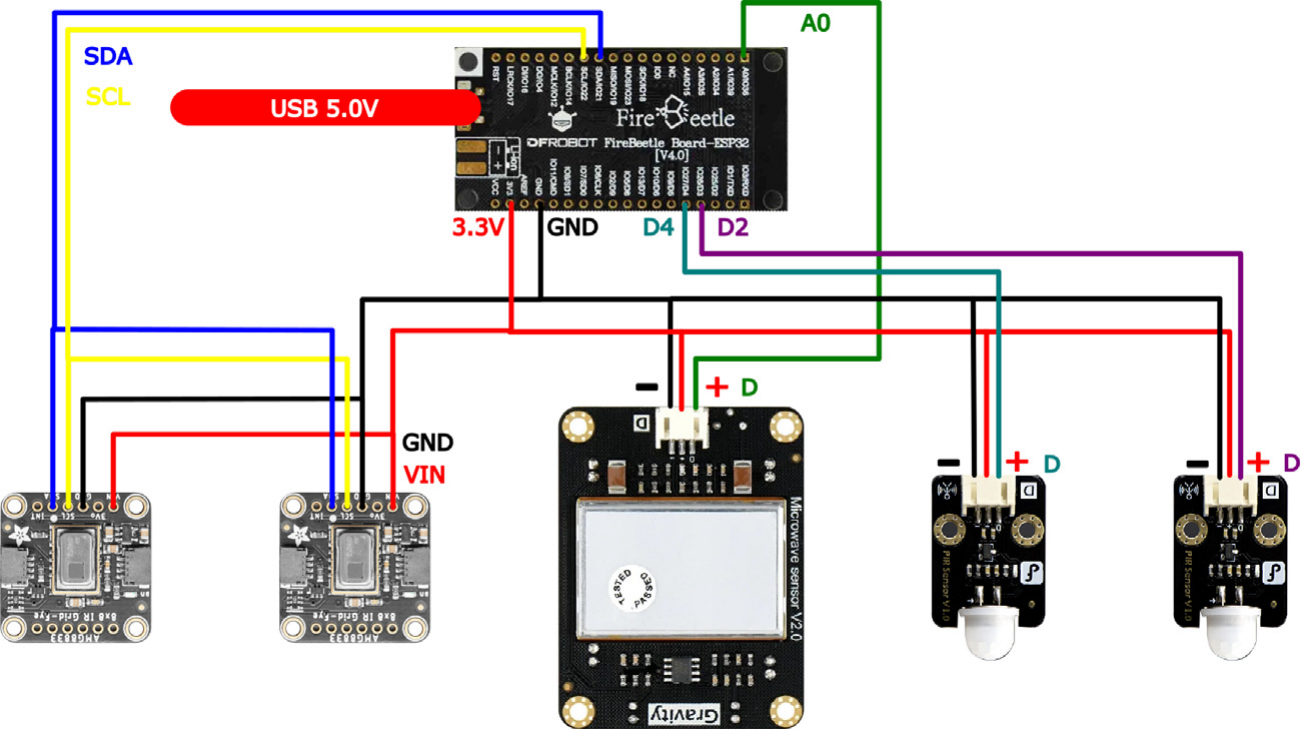
Connection diagram of the designed system.

**Fig. 4:**
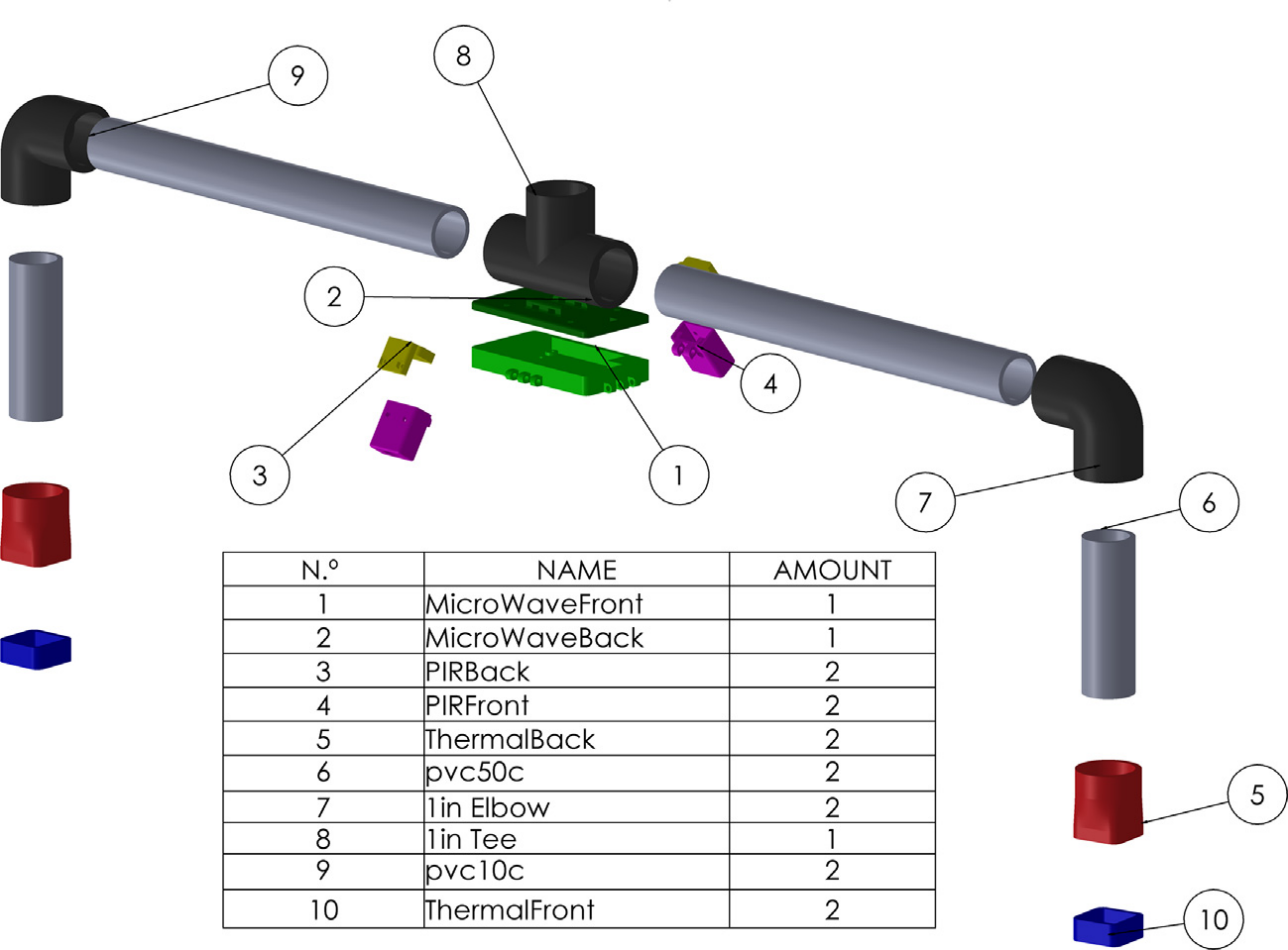
Exploded assembly of the system with the list of required mechanical parts.

The MCU was fixed in this version using plastic ties because the system can be used with other acquisition systems and other cards to give it greater versatility. Note that some of the steps described above can be executed in different order without altering the final result. However, for convenience for replication, we recommend you follow this step by step.

## Operation instructions

For the use of the system, some factors must be taken into account, such as:

• The sensitivity of the microwave sensor at its maximum power can deliver different readings about the same individual. However, in case its sensitivity is at a minimum, it will be difficult to detect human beings in very open environments or when the system installation is very far from the subject area.
• The system must be installed in the frame of a threshold or door, this because its sensors are oriented to help determine the direction of travel of the individual.
• The system is not developed for outdoor operation. However, by applying electronic coating you can protect the system from these effects.
• For system fastening, use 3/4 inch PVC pipe, plastic ties, or 3/4 inch metal clamps and screws to anchor it to a wall according to the installation situation.
• After system installation, the MCU must be connected to a 5 V and 2A max power supply. Power supplies with low power can generate errors in the reading of the sensors. Once connected, the system will begin to capture the movement data of the environment, returning via Bluetooth the information of each sensor when the movement of an object or individual is detected. Note that this information can be used not only for the control of some auxiliary system (counting or not) useful for the development of smart cities but also for the training or validation of artificial intelligence systems.

## Validation and characterization

**Fig. 6:**
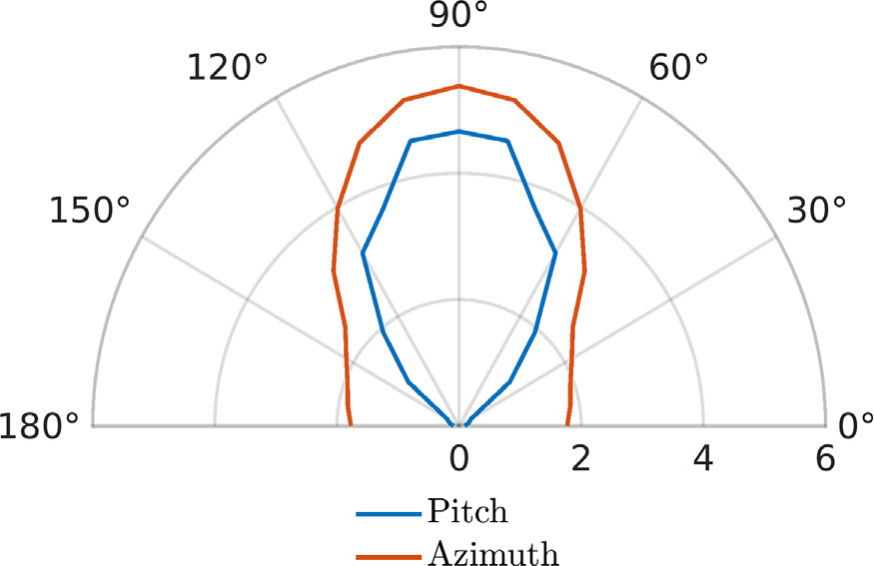
Polar graph for detection range of the PIR sensor in meters.

**Fig. 7:**
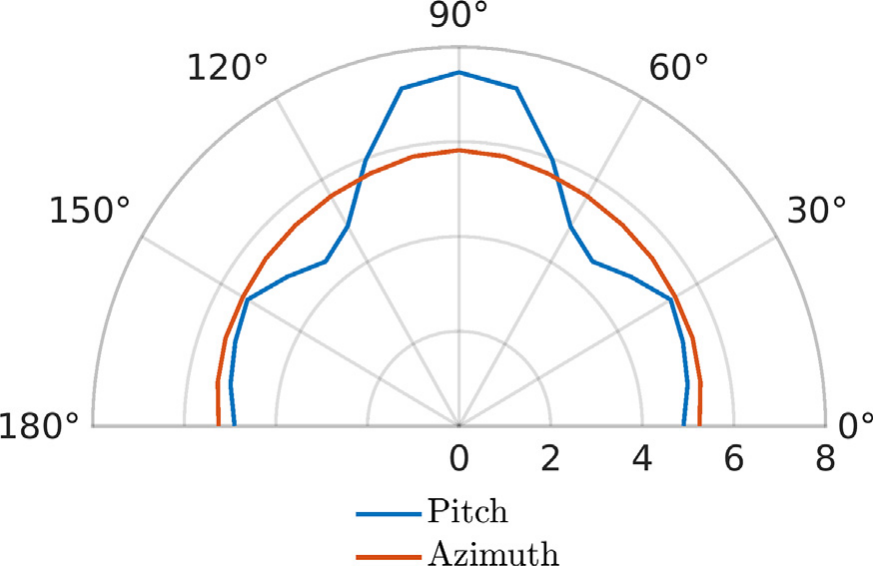
Polar graph for detection range of the MicroWave sensor in meters.

Range and accuracy validation of PIR and MW sensors was carried out by installing each detector at a 1.5 m height in a soccer field without people and obstacles. Tests were carried out verifying the detection distances of some pedestrians with a laser meter and plotting them into a polar graph. The experiment was repeated four times and the results were averaged to have a more realistic measure. Additionally, the averaging was made symmetrical to obtain the cone and the accuracy of each sensor for the quadrants of interest (I and II). From this, the following Fig. 7, Fig. 6 are obtained, which show the range of effectiveness of the previously mentioned sensors.

Since the system is based only on the sensors to determine the presence of movement, and filters adjust the range of speed and frequency of this we can say that the overall accuracy is the same as that of the sensors. Tests after adjusting the filters showed that there are no false positives in the system since there are no moving bodies of water for indoor applications that may affect the readings. In addition, the system indicates the presence of pedestrians after their appearance in the coverage area of this without random activations or noise. In case of any disturbance, the filters must be checked to adjust the operation of the systems.

Later, the system was evaluated using visual validation, employing a high-resolution video camera positioned at a fixed point, for the measurement verification concerning various actions or events sensed by the system. The system was tested with different pedestrian flow scenarios, and the movement of an unmanned quad-copter to simulate robotic platforms of various heights. It is important to clarify that, although the drone size is not variable, by changing the distance at which it is presented to the system, the area it occupies from the perspective of the sensor increases. These tests show the differences in the system response in the presence of mechanical systems or humans, which is a valuable feature for collaborative robotics environments.

**Fig. 8:**
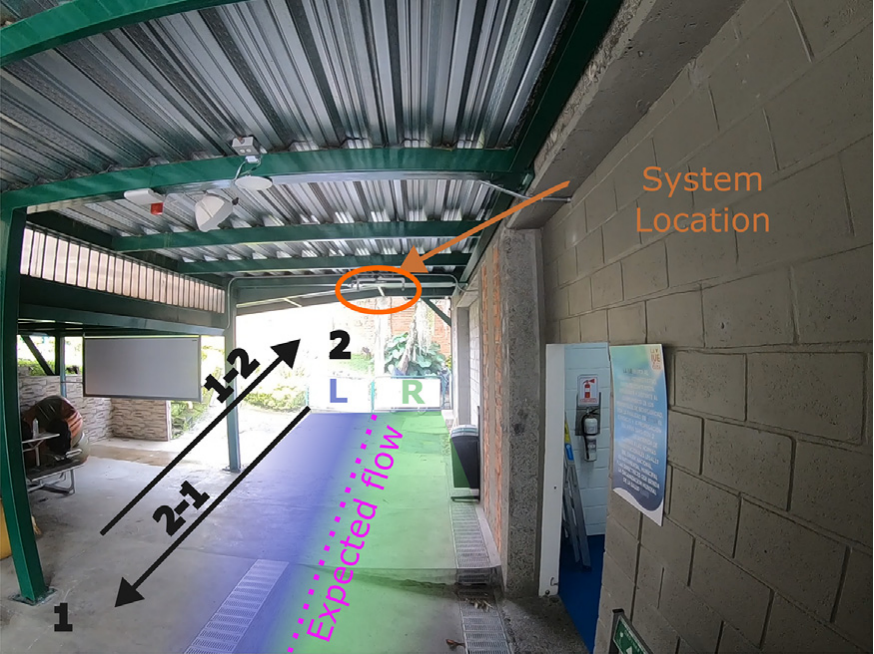
System location, analyzed directions, expected pedestrian Flow marked with purple doted lined, system right side with green, and left side of the system with blue.

The system validation was deployed in semi-controlled conditions within the Universitary Institution of Envigado (IUE). Although system installation was carried out in an open environment, the system was protected by an aluminum-clad concrete roof that protects it from the weather and isolates the microwave sensor from non-desired measurements. The system is positioned at approximately 3 m of height and fixed using plastic ties, taking special care with the system level so that the capture is orthogonal to the ground plane, with the sensitivity of the microwave sensor adjusted to the maximum. During the installation stage, the user must calibrate the system according to the study area, taking into account that the smaller the area or distance of coverage, the lower the sensitivity required for the detection of a human being. Caution is advised when installing the Microwave sensor, it manages to sense the back of the sensor as seen in the Fig. 8 and can penetrate objects, and some walls, use an aluminum insulator to avoid readings outside the interest area when necessary.

**Fig. 3:**
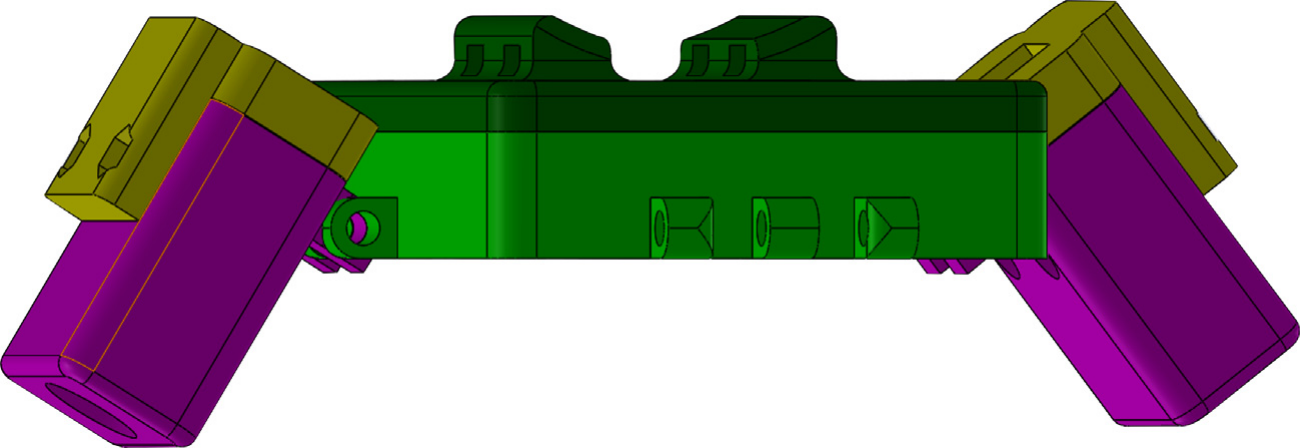
Hinge detail and Pir recommended orientation.

The PIR1 and PIR2 sensors were arranged to indicate the direction of the pedestrian flow in the expected transit axis and should point as shown in the Fig. 3 to increase detection range. Additionally, the developed C code has two filters that discriminate false positives and walking speed. Since infrared sensors undergo multiple activation in short periods of time due to the presence of an individual and even isolated activation due to noise, a frequency filter was applied to establish the presence of movement, if the number of readings is less or equal than 8 the system will reject false isolated readings and determine when real motion occurs if it is greater. This filter can be controlled in the movement() function where the difference between the reading frequency and the number of readings is set to 8. At line 69 ”abs(count - lastcount) > 8)”.

**Table:** 

bool movement() {
if ((dataMW == 1 or datapir1 ==1 or datapir2 ==1
or datapir3 ==1 or datapir4 ==1 or
(abs(tprev1-thermal1)>0.5) or (abs(tprev2-thermal2)>0.5))
and abs(count - lastcount) > 8) return true;
return false;
}

The second filter is a time filter that discriminates movement by its speed to detect in ranges of 200 ms, thus rejecting fast motion such as vehicles, thrown objects, or some animals. The filter can be modified by changing the delay at the end of the motion detection at line 99. As the delay decrease motion speed detection increases and vice versa.

**Table:** 

if (movement()) {
Serial.print("Movement Detected");
motioncount ++;
Serial.println(motioncount);
sprintf(buffer,
‘‘PIR1: %d -- PIR2: -%d -- MW: -%d -- TC1: -%f -- TC2: %f"
datapir1, datapir3, dataMW, thermal1, thermal2);
SerialBT.println(motioncount);
SerialBT.println(buffer);
lastcount = count;
delay(200);
}

The results show that the system logs between two to five events related to a movement for the configuration presented in this article. Such a number depends on the speed filter applied. Increasing the filter decreases the number of events detected by the system, while lower values will result in more events and even false positives. For the current installation, the study area is relatively large, so you have a speed filter equal to five and an event refresh rate equal to three seconds.

**Fig. 9:**
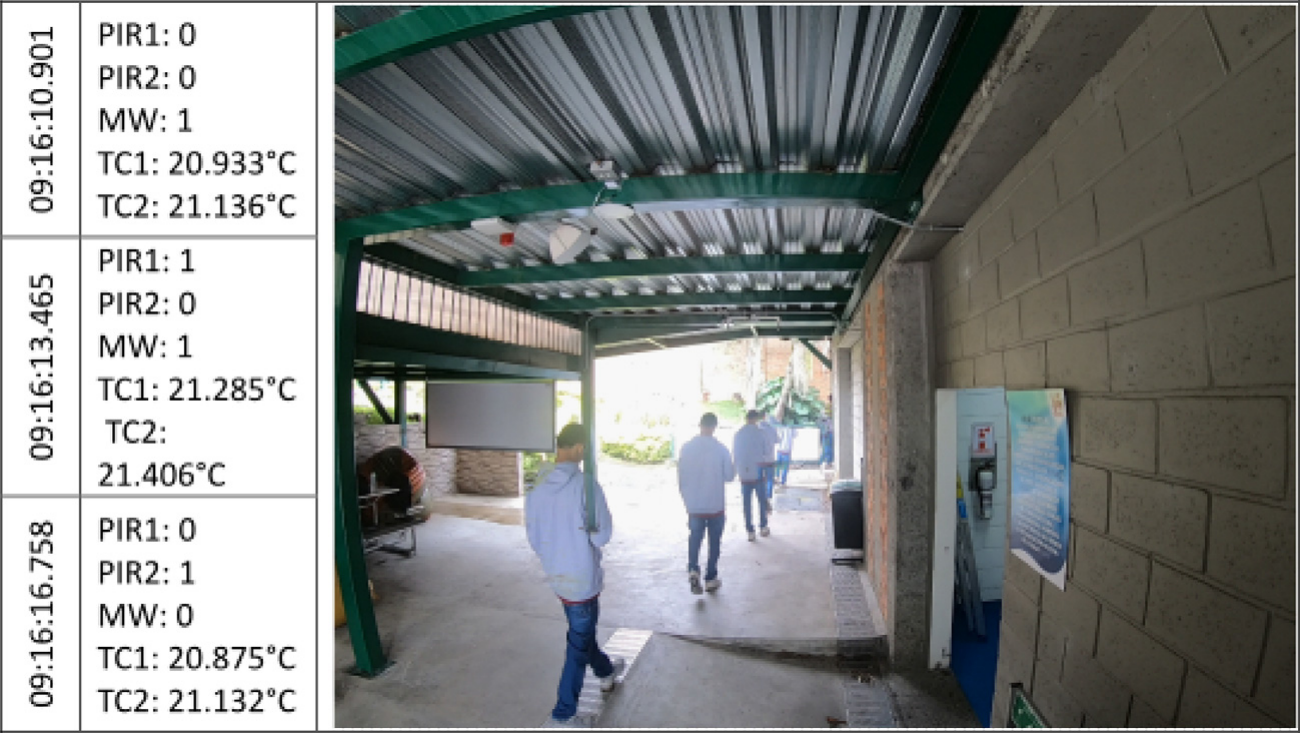
Central displacement in 1–2 direction, single human.

**Fig. 10:**
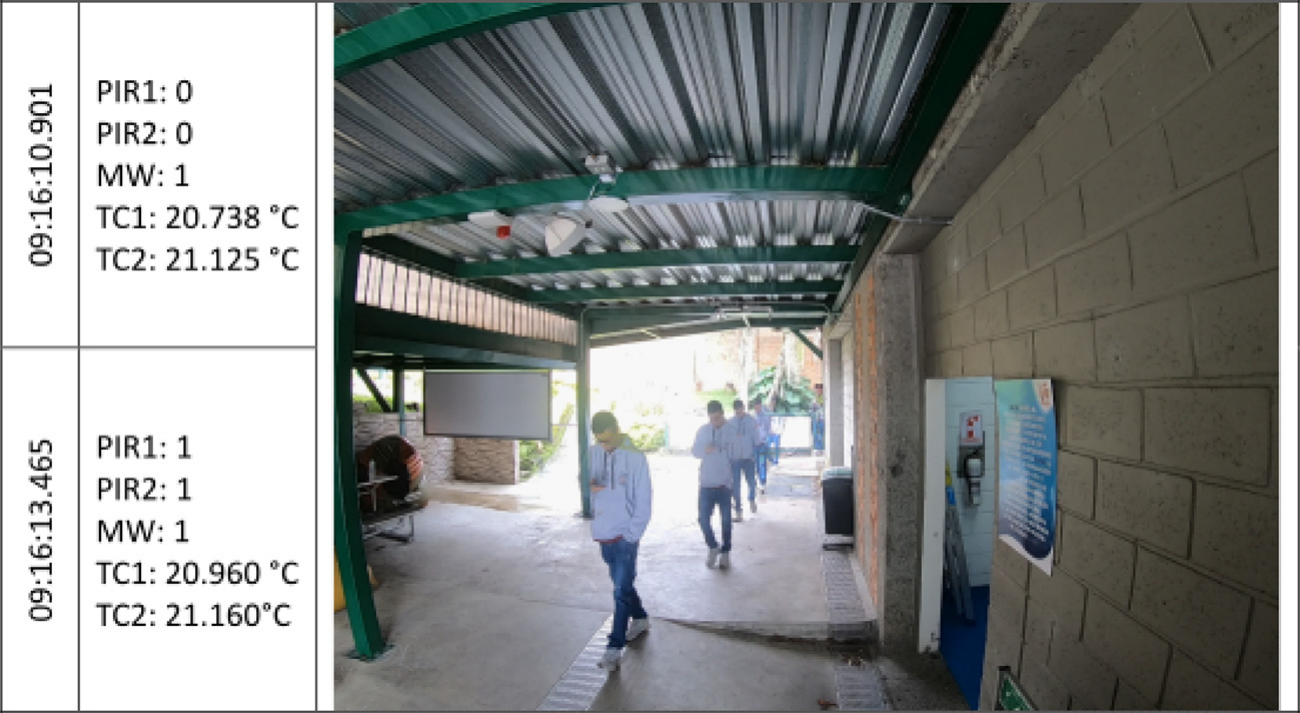
Central displacement in 2–1 direction, single human.

**Fig. 11:**
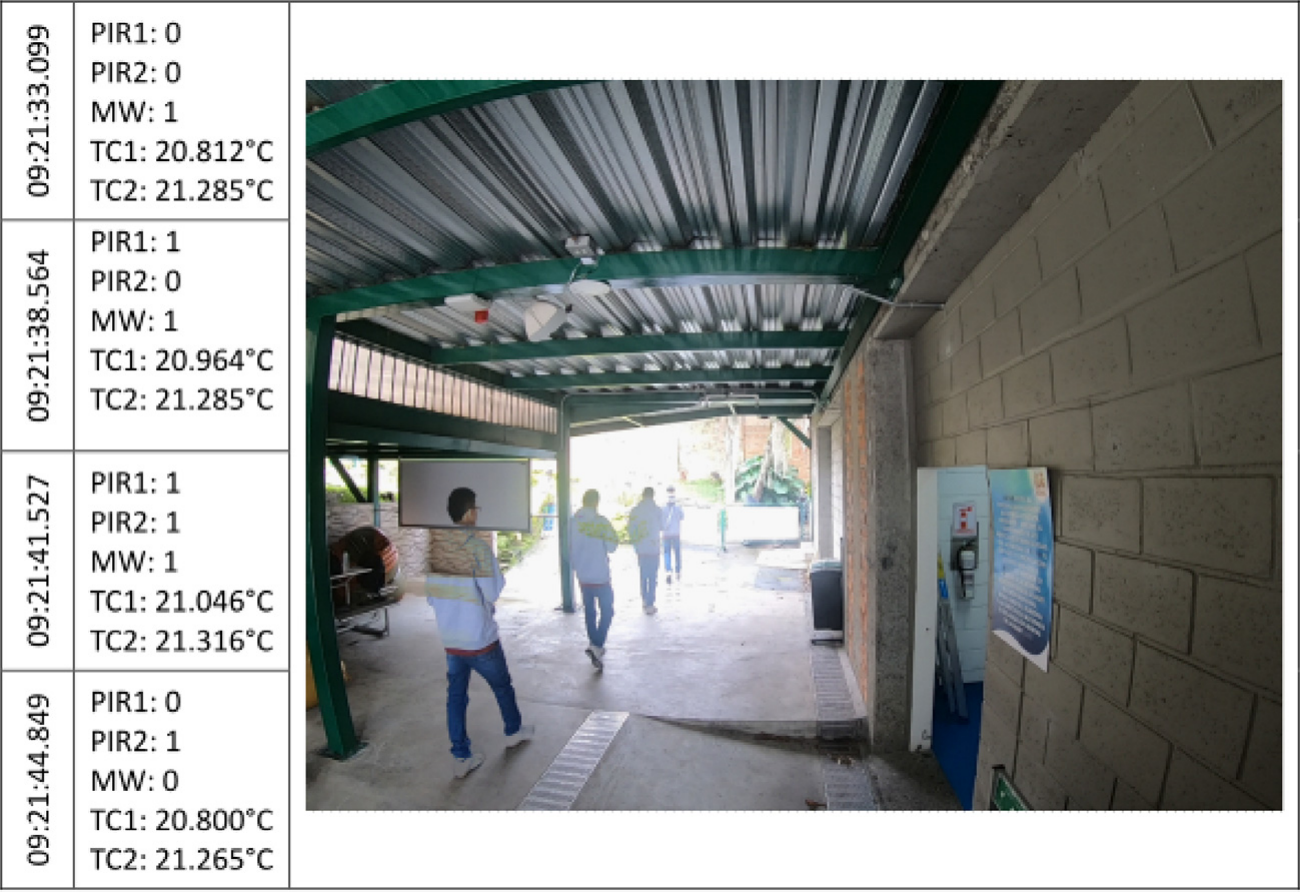
Left side displacement in 1–2 direction, single human.

**Fig. 12:**
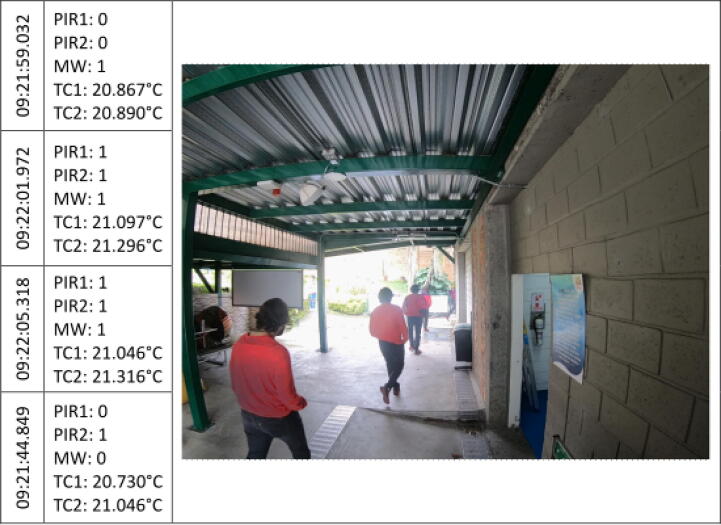
Right side displacement in 1–2 direction, single human.

**Fig. 13:**
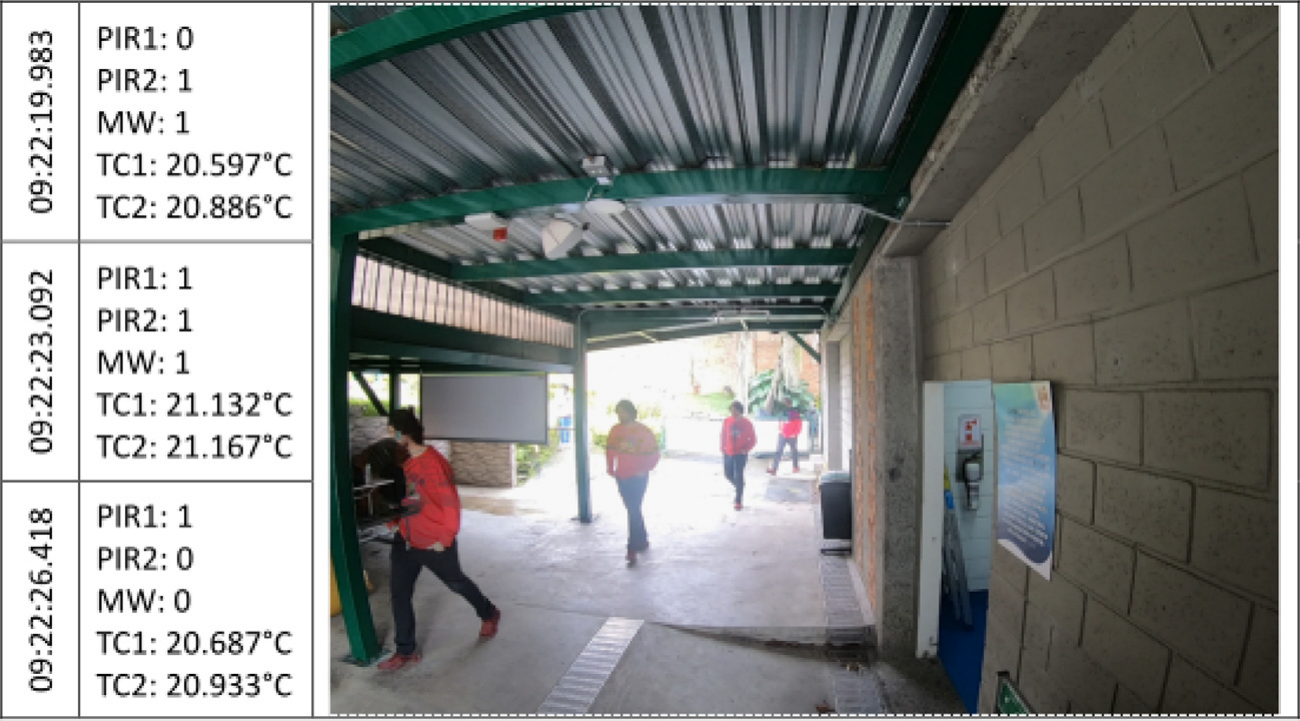
Right to left displacement in 2–1 direction, single human.

**Fig. 14:**
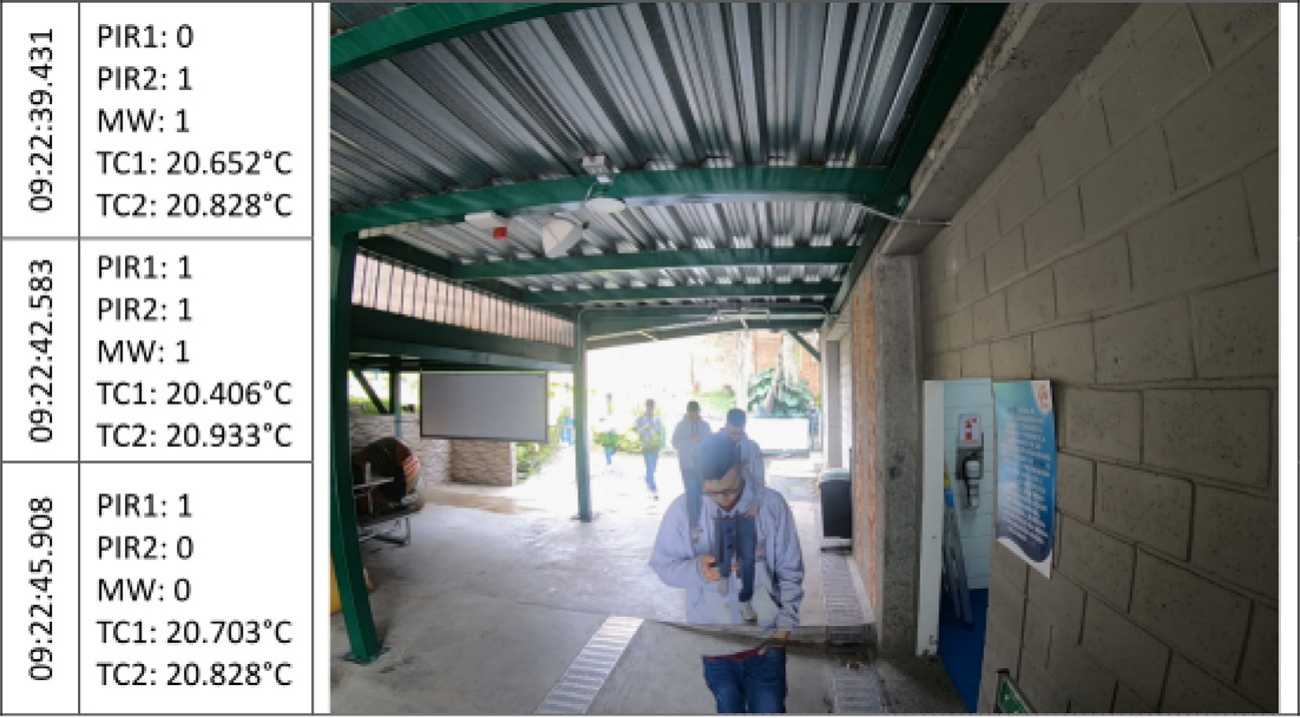
Left to right displacement in 2–1 direction, single human.

**Fig. 15:**
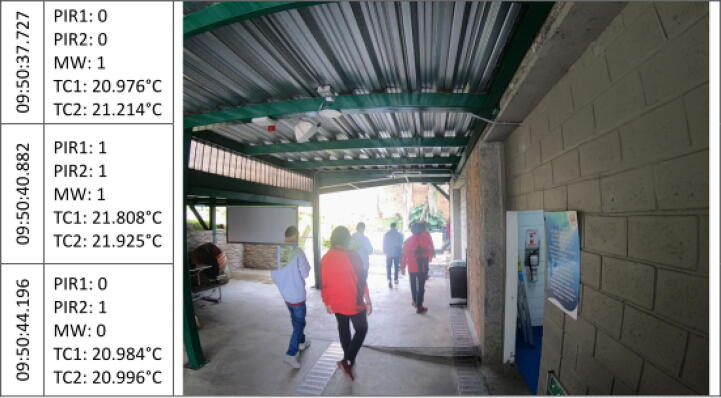
Displacement in 1–2 direction, pair of humans.

**Fig. 16:**
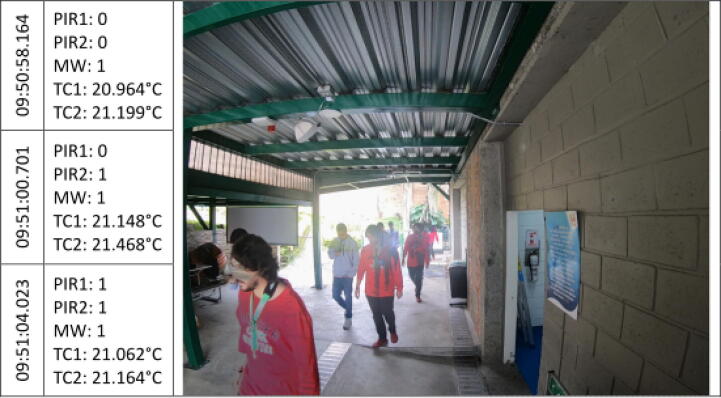
Displacement in 2–1 direction, pair of humans.

**Fig. 17:**
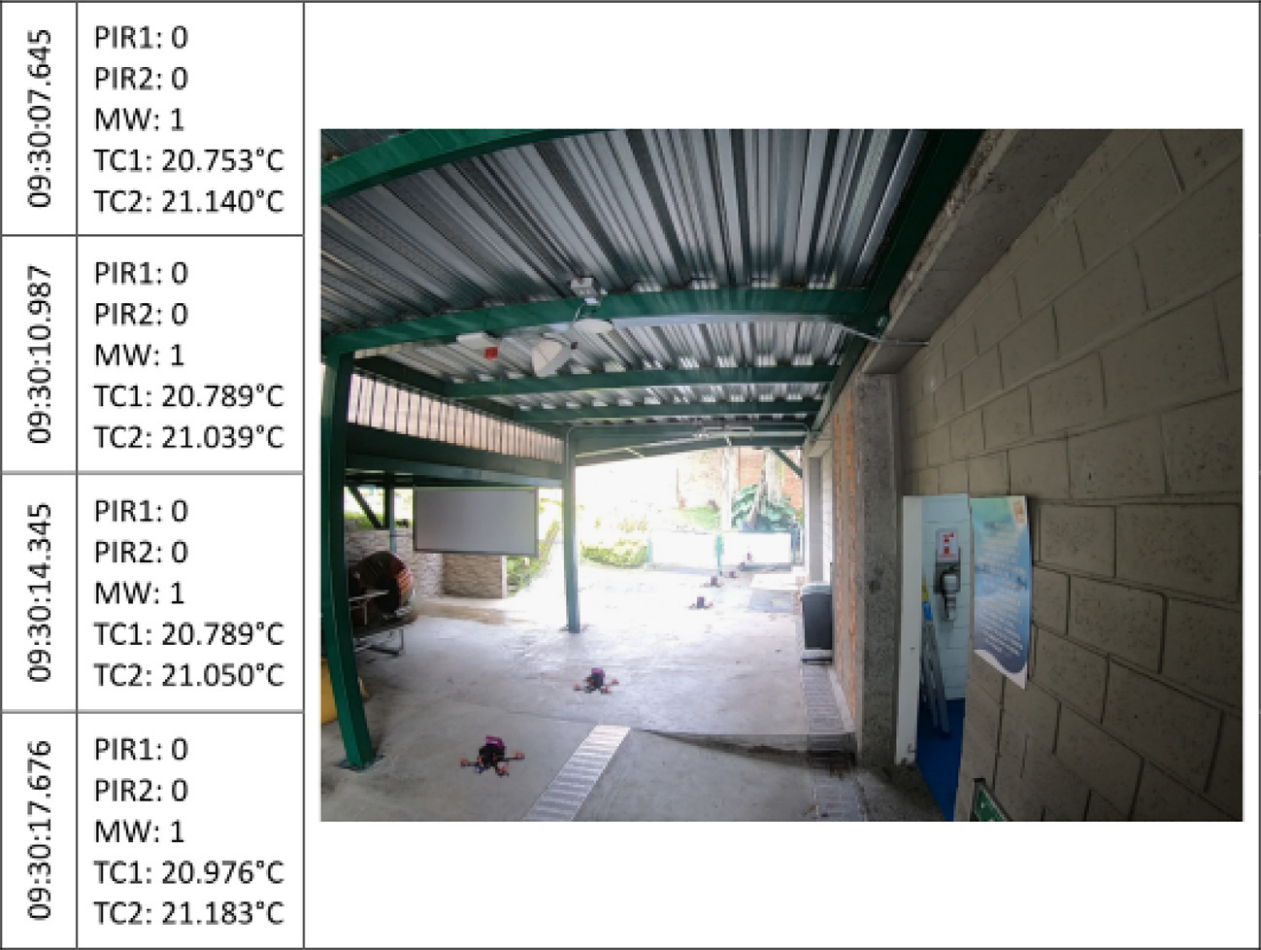
Displacement 1–2, Drone.

The Fig. 9, Fig. 10, Fig. 11, Fig. 12, Fig. 13, Fig. 14, Fig. 15, Fig. 16, Fig. 17 below reports performed tests, each depicting the timestamp in the left column, which the event related to the type of movement. The system is verified by video inspection. For the video capture, a GoPro HERO black 6 in 1440p @ 25 fps configuration was used and is represented by images that show the movement of the studio object every 50frames, that is, every 2 s for the captured video format. The movement type is shown using acronyms that indicate the activation of each of the sensors (1 for true, 0 for false), except for the thermal cameras that return the average temperature. PIR, MW, and TC are the infrared presence sensors, the microwave, and the thermal cameras respectively.

The following two Fig. 9, Fig. 10 show a measure of an individual’s gait centered in the system with directions 1–2 and 2–1 respectively. The figures show the real situation of the pedestrians who passed through the place where the data acquisition was carried out. The operation of the system was validated by comparing a video of crossing pedestrians with the information gathered from the sensors. The system detects the variation in the environment under the presence of people. The images show the pedestrian trajectory and the measures are given by the activations of the system which demonstrate related behaviors depending on pedestrian movements.s Fig. 9 shows that the direction of travel is distinguished in the infrared sensors, activating first the PIR1 and then the PIR2. And presenting a mid-event that shows the MW and the PIR1 activation. Nevertheless, the sensors did not trigger at the same time for this shot.

In the Fig. 10, the individual walk in the opposite direction to the previous one (2–1). A key crossing event is differentiated in which both PIR sensors and MW were triggered. Besides, a slight increase in the TC1 temperature related to a bias to the right in the subject path.

In the Fig. 11, the subject moves under the left side of the prototype. The system registers an event with the activation of the PIR1 sensor, followed by the activation of MW, PIR1, PIR2, and a temperature increase in the TC1 that represents the subject presence on the left side of the system. Finally, the motion ends with the activation of PIR2 which denotes the movement direction.

The before mentioned exercise was repeated in Fig. 12, but walking on the right side of the system in 1->2 direction. In this case, the displacement direction of the individual was not evidenced. Nevertheless, both PIR and MW were triggered indicating the presence of a person. In addition, there is a significant increase in TC2 compared to TC1, which corresponds with the subject walking. The temperature change is approximately 0.3 degrees Celsius, both sensors raise their measured temperature in the moment of pedestrian crossing and, taking into account correlation with the other sensors, the change in the environmental variables in the experiment is notorious. The temperature change is measured with respect to the average of previous measurements. Every time there is a considerable variation in temperature, the system activates a pedestrian count triggers.

The following Fig. 13 presents routes 2–1 in the right to left side direction. The flow direction was evidenced by the activation of PIR2, followed by the simultaneous trigger of PIR1 and MW. Since the walking path was a diagonal displacement, an increase in the temperature value is also evident in both TC.

Fig. 14 shows the direction of travel 2–1 with diagonal displacement from the left side to the right side. Although the system demonstrates the subject track successfully, observed temperature variations were minimal. Such behavior could occur because the route was not made just over the sensors and presents a bias in the displacement, this factor must be taken into account during the installation, since narrower entryways such as doors or corridors, can decrease this effect.

Additionally, tests are performed with the direction of travel 1–2 Fig. 15 and 2–1 Fig. 16 for a pair of individuals who cross the threshold to establish the robustness of the system. The results obtained were similar to those demonstrated above.

Finally, a series of tests were carried out with a drone at different speeds and heights. All the results present the same behavior where only the MW triggers, for this reason only one of the results stands in this paper, see Fig. 17. Tests were done with additional objects such as water bottles with the same results as for the drone, only the MW sensor activates, and the TC variations were insignificant.

• A thorough system installation is crucial to improve the accuracy of the acquired variables. However, given the system attributes and the obtained results, its functionality is reliable for assisting pedestrian flow analysis tasks.
• The system demonstrates the ability to differentiate objects from humans using the different features delivered by the system (PIR, MW & TC). Such information can lead successfully to the support of computer vision systems or other mechanisms implemented for smart cities.
• As future work, this project aims to create a database with the information acquired by the system for the training of a model that allows distinguish human beings from machinery or objects within collaborative robotics environments.

## Future work

The acquisition of data is proposed not only with people but also with other types of living beings and mobile objects, where the system can differentiate the variations with respect to each one, mitigate false positives and other possible disturbances. The use and storage of the acquired data will be used in the future for the training of a machine learning algorithm. The database that will feed the system will be located in a cloud storage service where information can be verified and shared in order to aim at the development of smart cities.

## Ethics statements

No human or animal studies were conducted in this work. The involved human subjects have given his informed consent.

## CRediT authorship contribution statement

**M. Mejia-Herrera:** Software, Investigation. **J.S. Botero-Valencia:** Methodology, Conceptualization, Validation. **D. Betancur-Vásquez:** Writing – original draft, Visualization. **E.A. Moncada-Acevedo:** Project administration, Supervision, Writing – review & editing.

## Declaration of Competing Interest

The authors declare that they have no known competing financial interests or personal relationships that could have appeared to influence the work reported in this paper.
